# Management of Steroid-Induced Diabetes: A Review of Herbal Medications and Their Role in Modulating Insulin Signaling Pathways

**DOI:** 10.2174/0118715303327497241214054628

**Published:** 2025-05-23

**Authors:** Jitendra Gupta, Neha Verma, Ankita Wal, Krishna Chandra Panda, Vishnu Vandana Yeleti, Bhupendra Singh, Shriya Mahajan, Harsimrat Kandhari, Pranay Wal

**Affiliations:** 1 Institute of Pharmaceutical Research, GLA University, Mathura, Pin Code 281406, U. P., India;; 2 Department of Pharmacy, PSIT-Pranveer Singh Institute of Technology (Pharmacy), NH19 Kanpur Agra Highway, Kanpur UP, India;; 3 Department of Pharmacy, Roland Institute of Pharmaceutical Sciences, Berhampur, Odisha, India;; 4 Department of Pharmacy, Avanthi Institute of Pharmaceutical Sciences, Vizianagaram, India;; 5 School of Pharmacy, Graphic Era Hill University, Dehradun-248002, India;; 6 Department of Pharmacy, S.N.Medical College, Agra-282002, India;; 7 Centre of Research Impact and Outcome, Chitkara University, Rajpura-140417, Punjab, India;; 8 Chitkara Centre for Research and Development, Chitkara University, Himachal Pradesh-174103, India

**Keywords:** Glucocorticoids, steroid-induced diabetes, herbal drugs, hyperglycaemia, insulin resistance, hepatic gluconeogenesis

## Abstract

**Background:**

Steroids are pharmaceuticals that have been extensively used for the treatment of numerous medical ailments. However, they have several negative effects, one of which is hyperglycaemia or Steroid-induced diabetes. It may happen to anybody, with or without a history of diabetes.

**Objective:**

The core objective of this review is to elucidate the pathogenesis, clinical risk factors, and significance of different types of herbal medications utilized in managing steroid-induced diabetes among patients undergoing glucocorticoid therapy.

**Methods:**

The relevant data was obtained by reading many sources, including review papers from various publications that included keywords like glucocorticoids, hyperglycaemia, steroids, and herbal medications. Additionally, information was gathered from online sources.

**Results:**

Steroid-induced diabetes mellitus (SIDM) represents a typical side effect stemming from an uncontrolled elevation of blood glucose level and it is closely related to the long-term damage and dysfunction caused by steroid use. The most commonly used medicinal plants to help manage blood glucose include *Anethum graveolens, Gynura procumbens, Inula racemosa*, and *Gymnema Sylvestre*, and the predominant mode of action for many herbal products and secondary metabolites employed in the management of steroid-induced diabetes revolves around modulating insulin signalling pathways.

**Conclusion:**

Glucocorticoids may provoke hyperglycaemia by increasing insulin resistance, which increases hepatic gluconeogenesis while decreasing glucose absorption by peripheral tissues including muscle cells and adipocytes. Presently no current optimal treatment for steroid-induced diabetes is available. Herbal medications have been shown to effectively manage blood sugar levels by maintaining a steroid concentration.

## 1 INTRODUCTION

Steroids constitute a class of medications that boast a lengthy record of therapeutic success and are extensively used to manage an extensive array of health conditions, ranging from acute to chronic disorders [1]. Steroids have been used for decades to treat a variety of medical conditions, including autoimmune diseases, allergies, and certain types of cancer, due to their ability to reduce inflammation and suppress the immune system. At high doses, steroids serve as a cornerstone of therapy for various inflammatory illnesses by inhibiting the synthesis of pro-inflammatory cytokines, hindering T-cell activation, and reducing Fc receptor expression. Consequently, they trigger anti-inflammatory and immunosuppressive mechanisms, making them indispensable for the treatment of a diverse range of inflammatory conditions [2, 3]. Cortisol, a glucocorticoid hormone produced by humans, is vital for survival during periods of high stress [4]. Glucocorticoids exert regulatory effects on both innate and acquired immune systems. They influence the inflammatory response by preventing leukocytes from leaving the circulation and reaching the sites of infection or tissue damage. Additionally, glucocorticoids impede macrophage phagocytic activity and limit the production of inflammatory cytokines, both of which are necessary for the immune system to activate an inflammatory response. Furthermore, GCs induce T-cell depletion, resulting in a reduction in the activity of the acquired immune system, whereas GC therapy has no effect on B-cell function [5]. Currently, oral glucocorticoid prescriptions are relatively uncommon, with less than one percent of the global population receiving them. However, among individuals aged 70 to 79, this percentage increases to 2.5 percent [6, 7]. Since dexamethasone, a potent glucocorticoid medication has been demonstrated to be an effective therapy for patients with severe COVID-19, the use of glucocorticoids has become more prevalent during the pandemic. Although long-term glucocorticoid treatment may possess substantial therapeutic efficacy for managing inflammatory diseases, its effectiveness is limited due to a broad range of side effects, including steroid-induced diabetes mellitus.

Glucocorticoids contribute to the development of various conditions, including insulin resistance, central obesity, lipid disorders, high blood sugar levels, and fatty liver. Glucocorticoids have also been linked to an increased risk of cardiovascular disease, as well as bone loss and osteoporosis. One of the most well-known unfavourable outcomes of hyperglucocorticoidism is diabetes which may also be referred to as steroid-induced diabetes [8] (Table **1**).

A report [9] suggests that the utilization of oral glucocorticoids is associated with approximately 2% of new cases of diabetes mellitus globally. In healthy individuals who received glucocorticoids over 1 to 7 days, impaired insulin tolerance and disrupted glucose metabolism were observed. Furthermore, studies have shown that even a single dose of glucocorticoids can result in a significant increase in blood glucose concentrations in individuals with or without pre-existing DM [10, 11]. Glucocorticoids exacerbate hyperglycemia in a dose-dependent manner, and this effect occurs regardless of whether the patient has a history of DM [12, 13]. According to the findings of a retrospective study, after receiving corticosteroid treatment, 56% of hospitalized patients who did not have a previous diagnosis of DM encountered at least one instance of hyperglycemia, denoted by blood glucose levels > 200 mg/dl. The frequency of newly diagnosed glucocorticoid-induced diabetes mellitus fluctuates between 15% to 40% among patients receiving glucocorticoid therapy for conditions including inflammation of the joints, major transplants, malignancy, breathing problems, and kidney issues. In addition, a meta-analysis found that the incidence of glucocorticoid-induced hyperglycemia is less than 32%, while the incidence of DM is 19% in patients who were treated with glucocorticoids for more than one month. These patients did not have diabetes mellitus before receiving treatment with glucocorticoids [14].

Extensive utilization of steroids and their intimate correlation with high blood sugar levels and DM There is a lack of solid knowledge of the processes that are at the root of these deleterious effects as well as techniques to reduce and control them. Studies investigating the causes and therapies for reducing or preventing glucocorticoid-induced diabetes mellitus are ongoing and are anticipated to significantly improve the clinical outcomes for individuals using glucocorticoids [14].

Steroid-induced diabetes mellitus (SIDM) can develop as a side effect of corticosteroid therapy. SIDM is often reversible, particularly when the duration and dosage of steroid use are managed appropriately [15]. Herbal treatments may aid in the management of SIDM by addressing metabolic issues associated with steroid use, especially since steroids can induce inflammation that negatively affects insulin sensitivity. Herbs such as *Inula racemosa* and *Gymnema sylvestre* have been shown to effectively improve insulin sensitivity [16, 17].

There is evidence that the use of plants and herbal extracts has the potential to serve as a complementary approach to the management of diabetes that is caused by steroids. In the course of preclinical research, several plant species have shown encouraging findings in terms of their ability to reduce the hyperglycaemic effects of glucocorticoids. Extracts of *Anethum graveolens (dill*) [18] and *Gymnema sylvestre* [16], for example, have been shown to lower blood glucose levels in animal models of steroid-induced diabetes. However, there are some important factors to consider while using herbal medicines. While entire plant extracts may include numerous bioactive chemicals that work together, isolating individual molecules enables more accurate dosage and standardization. The complex structure of plant extracts may cause variations in effects and possible interactions. More thorough clinical research is needed to discover the best formulations, doses, and safety profiles for herbal therapies in steroid-induced diabetes. At the moment, herbal methods should be investigated [19].

## 2 RISK FACTORS

Beyond cumulative dosage and prolonged length of steroid course, proposed risk variables for steroid-induced diabetes include greater BMI, aging, familial susceptibility, and poor tolerance to glucose [20] Fig. (**1**).

The link between having diabetes in one's family and having diabetes in oneself has not been well established. Hwang and Weiss evaluated the clinical characteristics and demographic profiles of patients initially diagnosed with type 2 diabetes and those initially diagnosed with SIDM, considering whether the patients were receiving steroid medication at the time of the study. Compared with patients who had type 2 diabetes mellitus and were being treated with glucocorticoids, those who acquired new-onset steroid-induced diabetes mellitus were considerably less likely to have a family history of diabetes [21].

## 3 PATHOPHYSIOLOGY OF STEROID-INDUCED DIABETES MELLITUS

The diabetogenic outcomes of glucocorticoid drugs manifest through intricate mechanisms involving multiple physiological processes and organ systems as shown in Fig. (**2**).

Glucocorticoids have diverse impacts on glucose homeostasis, regulating numerous parts of the process. Glucocorticoids stimulate gluconeogenesis in the liver but reduce glucose absorption and utilization in skeletal muscle and white adipose tissue by inhibiting insulin responses [22]. The liver acts as a hub that connects dietary and hormonal stimuli to whole-body glucose homeostasis [23]. The liver is the primary organ for storing glucose in the form of glycogen as well as for endogenous glucose synthesis; hence, it plays an important role in maintaining glucose homeostasis [24]. During fasting, the liver facilitates euglycemia through gluconeogenesis and glycogenolysis, which are antagonized by insulin post-unkally. Glucocorticoids counteract the metabolic effects of insulin, specifically in the postprandial state, by inducing enzymes that increase gluconeogenesis [25].

### 3.1 Skeletal Muscle

In the postprandial state, skeletal muscle tissue emerges as the primary site for insulin-mediated glucose elimination, playing a crucial role in the metabolic processing of glucose [26]. Through a process known as glucocorticoid (GCs)-induced atrophy, GCs may cause total skeletal muscle mass to decrease, which in turn lowers glucose absorption in the skeletal muscle [27]. Steroids cause insulin resistance by directly obstructing signaling pathways, particularly those involved in the production of glycogen. Steroids promote protein degradation, resulting in higher blood amino acid levels that inhibit insulin distribution inside the muscle cells [28].

In addition to influencing skeletal muscle cells, glucocorticoids can also hinder glucose absorption by disrupting insulin-triggered mobilization of capillaries within skeletal muscle tissue. This may have resulted in impaired glucose uptake. This interference may reduce glucose uptake. According to the study, both low-dose (7.5 mg daily) and high-dose (30 mg daily) prednisolone treatments over two weeks exhibited a dose-dependent decrease in insulin-induced capillary mobilization in healthy people, as measured by capillary microscopy [29]. Glucocorticoids stimulate reductions in insulin, causing capillary mobilization, and are associated with alterations in fasting and postprandial blood sugar concentrations [30].

### 3.2 Adipose Tissue

There is a tendency for adipose tissue to encourage the deposition of fat in visceral areas while concurrently depleting peripheral reserves. Adipokines are directly affected by steroids in many ways, including: (1) They increase the expression of adipokines, which are pivotal players in regulating glucose tolerance. (2) Steroids prevent adiponectin expression, a key influencer of insulin sensitivity. (3) They stimulate the release and expression of leptin and increase lipid degradation in adipocytes [31].

Consequently, these processes lead to increased plasma concentrations of unsaturated fatty acids. These fatty acids accumulate in muscle cells and interfere with insulin signaling pathways, hindering the absorption of glucose [32, 33].

## 4 ISLET-CELL MALFUNCTION CAUSED BY GLUCOCORTICOIDS

The beta cells in the pancreas are very important for the process of glucose metabolism, and in recent decades, it has been understood that a malfunction in beta cells is the primary defective mechanism that leads to type 2 diabetes [34]. Under normal physiological conditions, insulin secretion has a hyperbolic relationship that is proportional to insulin sensitivity. This relationship was direct. The product of these characteristics, referred to as the disposition index, does not change [35]. Therefore, as the burden on beta cells rises (due to variables such as weight gain, resistance to insulin, or low-grade inflammation), healthy beta cells can be adjusted by increasing insulin production to meet the greater demand, thereby preserving euglycemia. This occurs when the beta cell is subjected to an increased workload. The inability of beta cells to release an adequate amount of insulin to fulfill the body's need for insulin leads to glucose intolerance and type 2 diabetes [36, 37].

## 5 HERBAL MANAGEMENT OF STEROID-INDUCED DIABETES

According to the World Health Organization (WHO), up to 80% of the population in the developing world relies on plants and their derivatives for essential healthcare needs. As per ethnobotanical information, 800 plants have been identified as having antidiabetic properties [38]. A diverse range of plant-derived active principles representing various bioactive chemicals has been identified for their potential application in the management of diabetes [39].

### 5.1 *Anethum Graveolens*

**Fig. (3):**
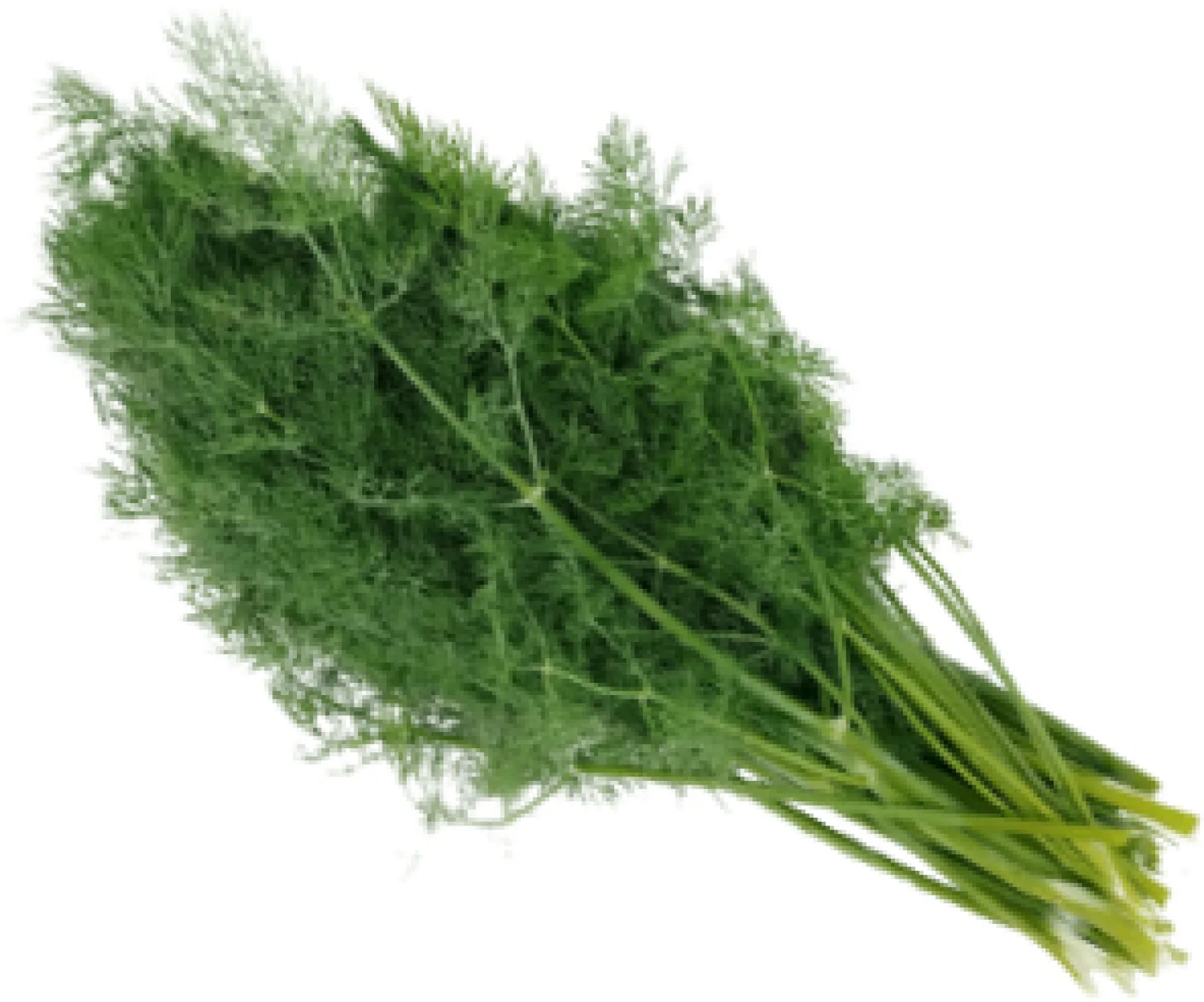
Herbal plant of *Anethum graveolens*.

*Anethum graveolens* L. is a prominent plant that is frequently used as a spice and produces essential oils (Fig. **3**). It has been used in Ayurvedic medicine for thousands of years. is a prominent plant that is frequently used as a spice and produces essential oils [40]. It has been used in Ayurvedic medicine since thousands of years. This herb has an anti-diabetic effect on steroid-induced diabetes. The effect of the leaf extract on the development of type 2 diabetes in female rats was examined.

In animals administered an equivalent dose of dexamethasone over a duration (22 days), the administration of leaf extract (100 mg/kg body weight/day) during the final 15 days resulted in a reduction in the concentration of both serum glucose and insulin. This observation underscores the potential efficacy of plant extracts for mitigating corticosteroid-induced diabetes [18].

As reported by Adisakwattana *et al*. [41], polyphenolic chemicals in plant extracts *Anethum graveolens* may have a significant role in inhibiting AGE development. Advanced glycosylation end product (AGE) production has been recognized as a critical factor in diabetic complications. AGEs have been linked to the development of oxidative stress and inflammation, both of which are associated with diabetic problems.

According to another study conducted by Takahashi *et al*. [42], *Anethum graveolens* extract regulated peroxisome proliferator-activated receptor-α (PPAR-α) and elevated the expression of genes associated with fatty acid oxidation, which corrected the lipid profile in diabetic obese mice. The finding also suggests that dill may possess compounds that enhance the solubility and bioavailability of glucose-regulating substances, potentially offering a natural approach to managing diabetes [43].

### 5.2 *Gynura Procumbens*

*Gynura procumbens* is a hypoglycemic agent Fig. (**4***). Gynura procumbens* contains bioactive compounds such as flavonoids and glycosides [44]. Kaempferol is a flavonoid that is present in *Gynura procumbens* and is slightly soluble in water but is substantially soluble in hot ethanol. The presence of flavonoids in this plant may contribute to its capacity to boost the solubility and bioavailability of insulin-sensitizing chemicals, hence increasing their efficiency in blood glucose control [45]. Kaempferol is a crucial antidiabetic micromolecule that enhances peripheral insulin responsiveness and protects against pancreatic cell failure.

Kaempferol was shown to improve lipolysis and prevent high fatty acid-impaired glucose uptake, glycogen synthesis, AMPK activity, and Glut4 expression in skeletal muscle cells when it was tested *in vitro*. By employing a different mouse model of type 2 diabetes, which was generated through the consumption of a high-fat diet and the administration of low doses of streptozotocin injection, we discovered that the administration of kaempferol resulted in a substantial improvement in hyperglycemia, glucose tolerance, and blood insulin levels in obese diabetic mice. These improvements are linked to the enhancement of islet β-cell mass. The findings of this study provide evidence that kaempferol has the potential to act as a naturally occurring anti-diabetic medication. It offers this by enhancing the sensitivity of peripheral insulin and guarding against pancreatic β-cell malfunction [44]. It has been demonstrated that *Gynura procumbens* extract has a limited antihyperglycemic effect in diabetic rats caused by steroid treatment in terms of live weight, and blood sugar [46].

### 5.3 *Inula Racemosa*

**Fig. (5):**
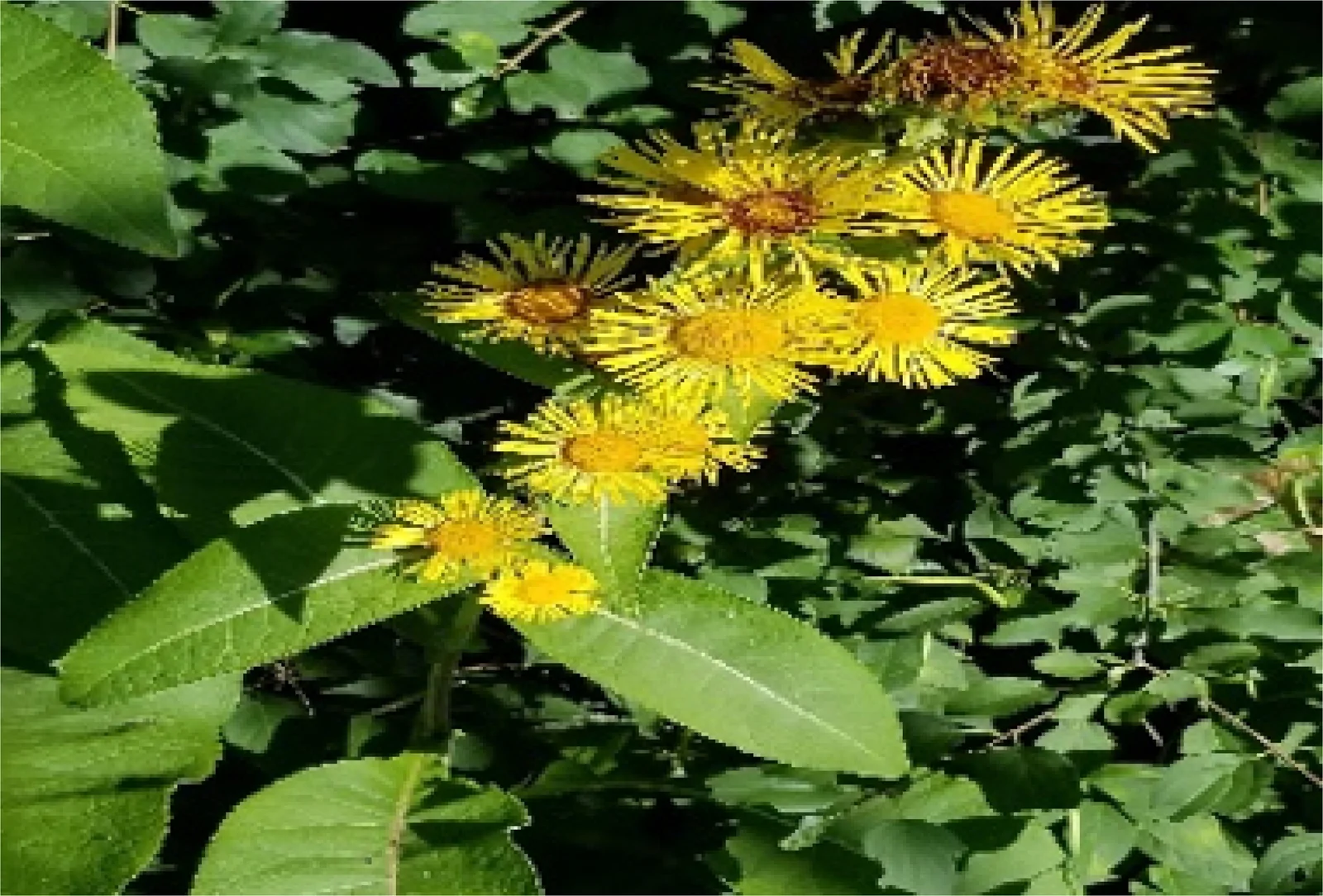
Herbal plant of *Inula racemosa*.

*Inula racemosa* is an Ayurvedic herb (Fig. **5**). It has been reported that it contains inulin, essential oil, sesquiterpene lactones - Alantolactone, isoalantolactone, isoalloalantolactone, and beta sitosterol, daucosterol, inunal, inunolide, and beta sitosterol [47]. A recent study found that *Inula racemosa*, along with two other plants, reduced blood cortisol and glucose levels. The hypoglycemic effects of the first three plant extracts appear to be mediated by their cortisol-suppressing abilities [48]. It has been shown that *Inula racemosa* lowers blood cortisol and glucose levels, most likely *via* cortisol-suppressive processes. The capacity of this plant to alter cortisol levels raises the possibility that it contains chemicals that improve the solubility and bioavailability of molecules involved in the control of stress hormones, which may have consequences for treating metabolic conditions associated with stress [48].

### 5.4 *Gymnema Sylvestre*

**Fig. (6):**
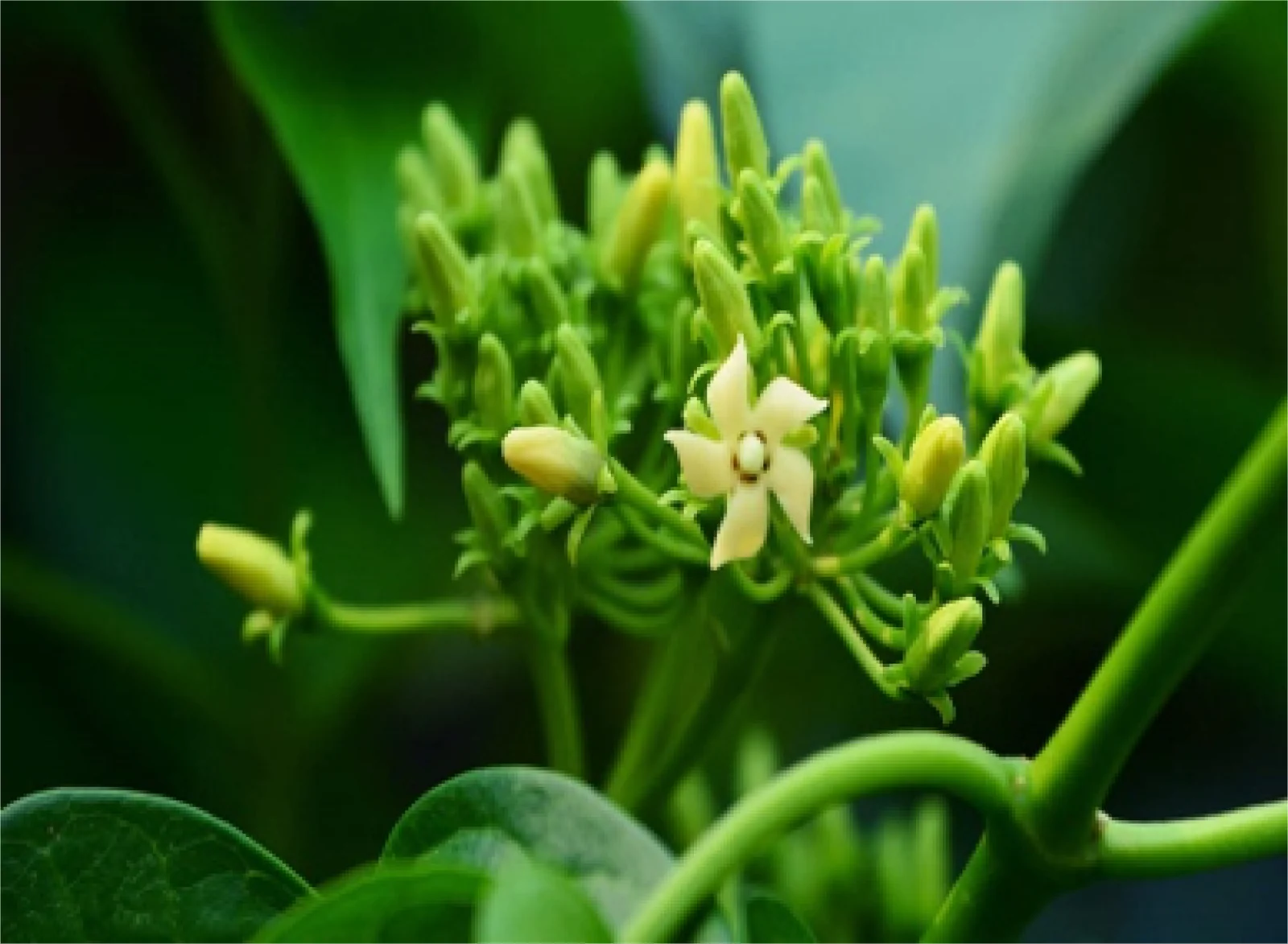
Herbal plant of *Gymnema Sylvestre*.

*Gymnema Sylvestre* is a member of the Asclepiadaceae family (Fig. **6**). It is a plant native to India's southwest, Australia, and Africa. Meshashringi is another name in Sanskrit. The active component of the plant is a class of acids called gymnemic acids [49]. It has been shown that there may be a connection between blood sugar levels, gymnemic acid levels, and adiposity. In one investigation, gymnemic acids were found to be therapeutic beneficial in the treatment of hyperglycaemia and SIDM. The gymnemic acids that are found in this plant may also have a function in enhancing the solubility and bioavailability of chemicals that are involved in the metabolism of glucose [50].

Another research found that *Gymnema Sylvestre*, alone or in combination with *Inula racemosa*, was useful in the treatment of SIDM in mice [51].

### 5.5 *Pterocarpus marsupium*

**Fig. (7):**
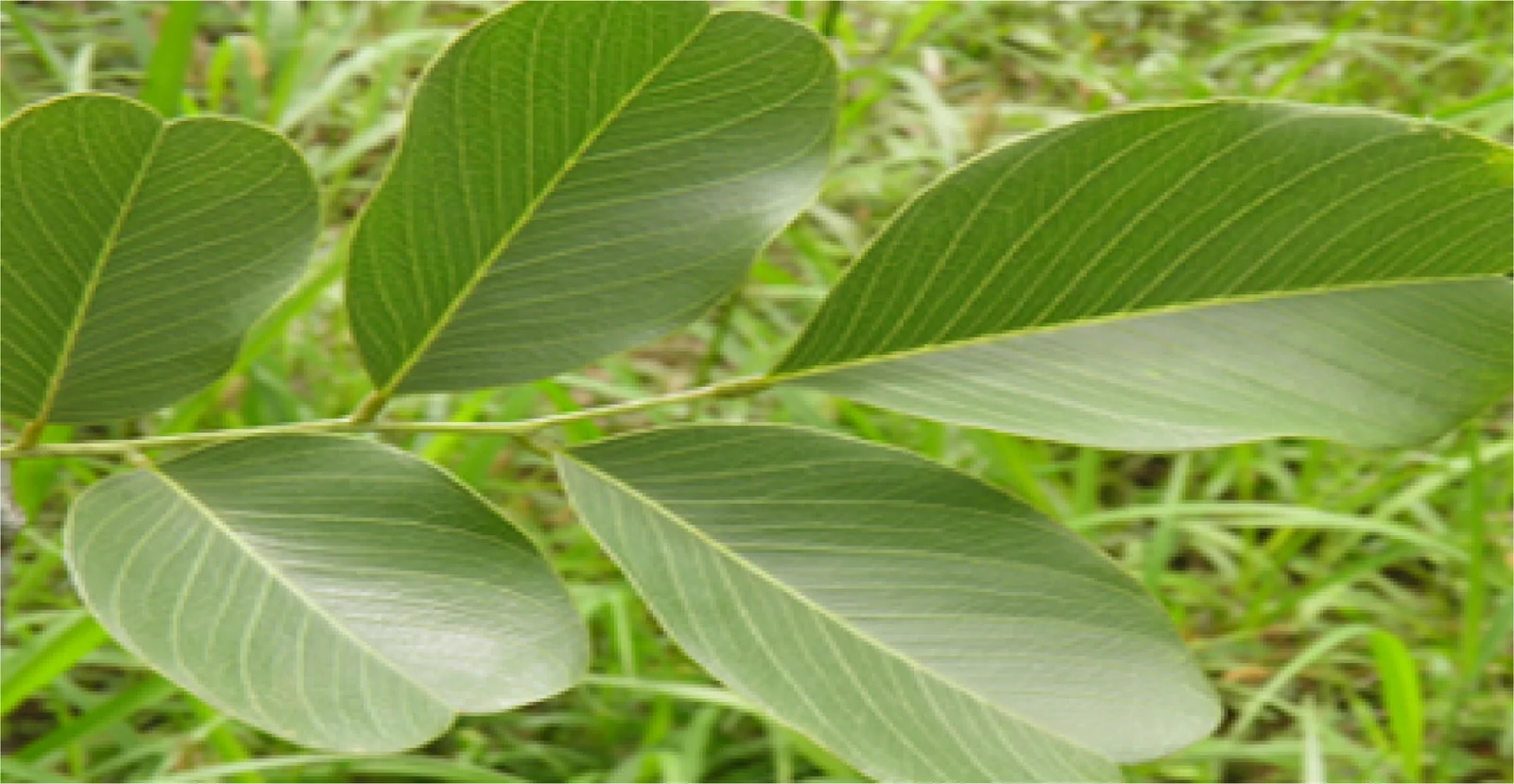
Herbal plant of *Pterocarpus marsupium*.

The Indian kino tree, or bijasar, is composed of *Pterocarpus marsupium*, a member of the Fabaceae family. Heartwood, leaves, flowers, bark, and gum are the most commonly utilized plant components [52] (Fig. **7**). In India, *Pterocarpus marsupium* grows to a great extent. Ayurveda is among the most adaptable therapeutic herbs and exhibits a diverse range of biological activities. The medicinal value of each tree component was determined. It is rich in polyphenols, β-sitosterol, lupenol, epicatechins, aurone glycosides, and isoflavonoids, among the phenolic and terpenoids found in *P. marsupium* [53]. We determined the medicinal value of each tree component. These are some of the phenolic and terpenoids that can be found in *P. marsupium*: isoflavonoids, β-sitosterol, lupenol, epicatechins, aurone glycosides, and polyphenols [54].

Researchers have demonstrated the usefulness of Pterocarpus in treating steroid-induced diabetes. In a preclinical study, the ethanolic extract from the heartwood of *Pterocarpus marsupium* lowered high blood sugar levels caused by dexamethasone by a large amount. The insulin-sensitizing qualities exhibited by *P. marsupium* surpassed the efficacy of pioglitazone, a commonly used diabetic drug. This discovery shows that the extract may include components that increase the solubility and bioavailability of insulin or other molecules that regulate glucose levels, which ultimately results in enhanced glycaemic management. This shows that the pterocarpus might be a useful natural alternative for controlling insulin resistance associated with steroid-induced diabetes [55].

### 5.6 *Costus igneus*

**Fig. (8):**
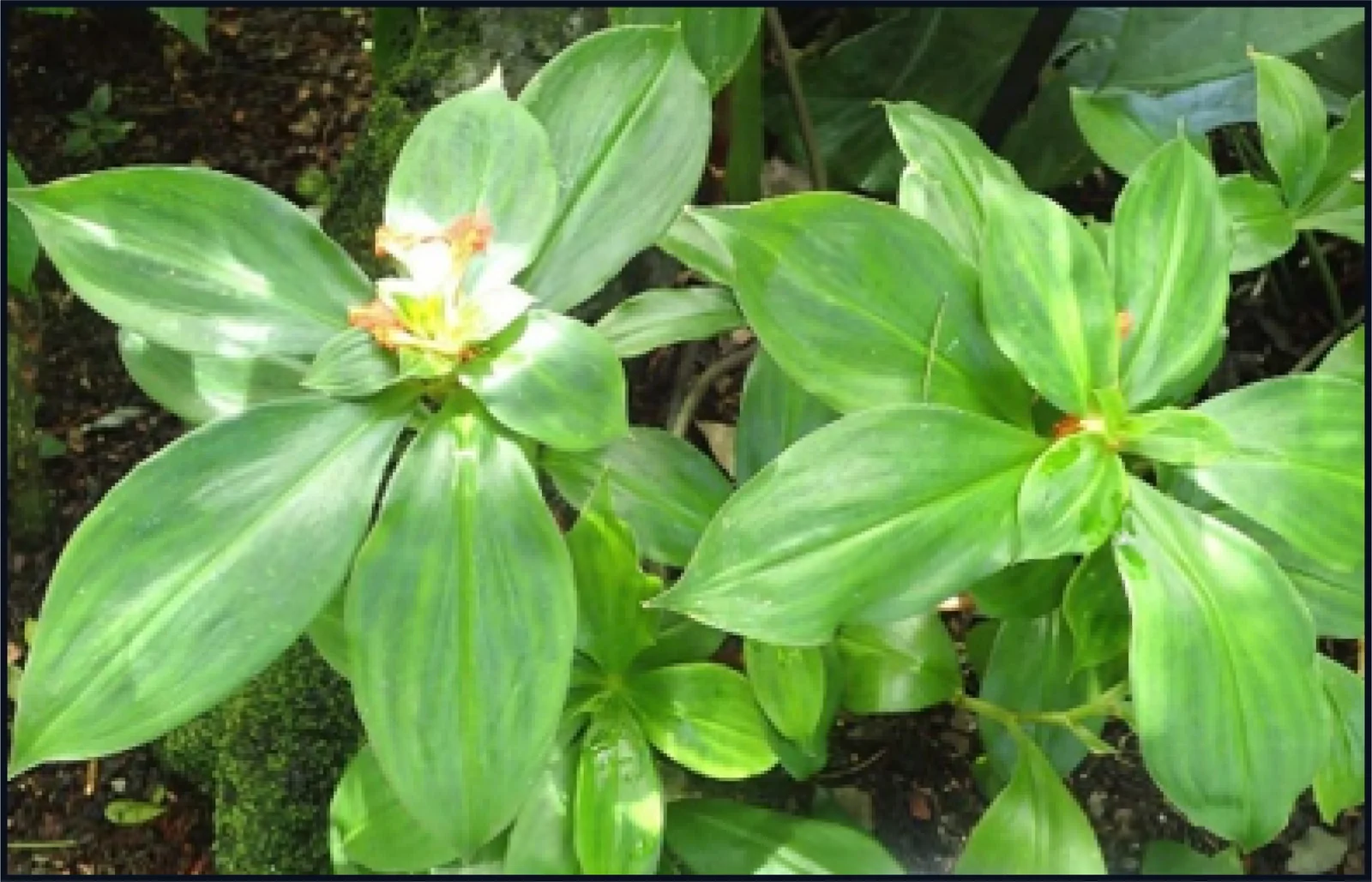
Herbal plant of *Costus igneus*.

In India, costus is recognized as an insulin plant because of its ability to increase insulin levels in the body [56]. People with diabetes consume one leaf every day to maintain low blood glucose levels. It has been used to treat diabetes in India (Fig **8**).

It is indigenous to Southeast Asia, particularly the Greater Sunda Islands in Indonesia, and is a recent introduction to India. It is predominantly cultivated as an ornamental species in the state of Kerala. Its natural habitat extends across Southeast Asia, where it thrives in a tropical climate. Despite its novelty in India, this plant has gained popularity owing to its ornamental value and potential health benefits [57].

Glucocorticoid-induced DM is similar to that caused by type 2 diabetes mellitus. A study was performed on male Wistar rats with dexamethasone-induced hyperglycemia. This study found that costus leaf powder lowered both fasting and blood glucose concentrations in rats with dexamethasone-induced hyperglycemia, returning them to normal levels. The active chemicals identified in *Costus igneus* have the potential to enhance the solubility and bioavailability of molecules that are involved in glucose homeostasis. This might provide a natural option for the management of hyperglycemia. As a result, it is useful in the management of SIDM [58].

### 5.7 *Elettaria cardamomum*

**Fig. (9):**
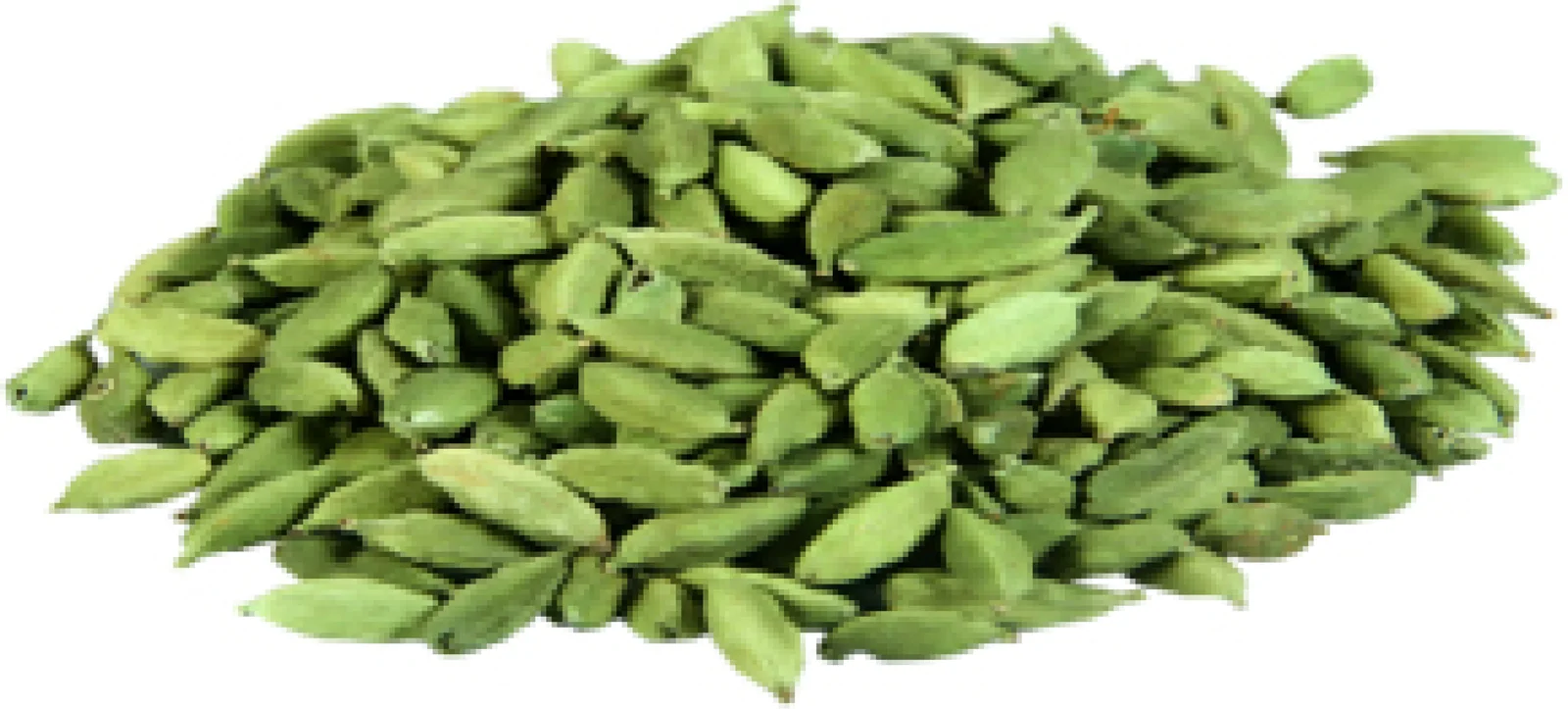
Herbal plant of *Elettaria cardamomum*.

Cardamom, famous as the “Queen of Spices, is an expensive spice in the world behind saffron. *Elettaria cardamomum* is the dried fruit of a perennial herb belonging to the Zingiberaceae family [59] (Fig **9**).

It contains high concentrations of phenolic compounds, volatile oils, and fixed oils. Its pharmacological effects include antihypertensive, antioxidant, hepatoprotective, hypocholesterolemic, and antidiabetic properties [60].

An experimental study was conducted involving albino rats that were orally administered a cardamom suspension (1 g/kg/10 mL of 2% gum acacia) for six days before and concurrently with the administration of dexamethasone. These findings indicate that cardamom considerably lowered hyperglycemia [61]. Cardamom (*Elettaria cardamomum*) suspension reduced hyperglycemia in dexamethasone-treated rats, perhaps *via* antioxidant and hypolipidemic actions. This implies that cardamom may include chemicals that increase the solubility and bioavailability of antioxidants and lipid-regulating molecules, which contribute to its favourable effects on glucose metabolism. Cardamom medication or dietary use may be beneficial in treating fasting hyperglycemia caused by glucocorticoid excess [62].

### 5.8 Differences in Herbal Treatment for Chronic Diabetes Mellitus (DM) and Steroid-Induced Diabetes Mellitus (SIDM)

The herbal management of chronic diabetes mellitus (DM) and steroid-induced diabetes mellitus (SIDM) differs because of the mechanisms, causes, and physiological reactions associated with each disease.

#### 5.8.1 Chronic Diabetes Mellitus

The therapy's primary focus is improving insulin sensitivity, boosting beta-cell activity, and treating related disorders such as obesity and metabolic syndrome [63]. Bitter melon, aloe vera, green tea, cinnamon, and fenugreek are examples of herbs that are often used in herbal therapies. These herbs have been shown to reduce blood sugar levels, enhance insulin sensitivity, and assist with weight control [64, 65].

#### 5.8.2 Steroid-Induced Diabetes Mellitus

Treatment for steroid-induced diabetes mellitus (SIDM) focuses on lowering inflammation and regulating blood sugar levels to mitigate the effects of corticosteroids, which are the causes of the condition. Because of the acute nature of steroids' effects, this often necessitates a more urgent treatment [66]. Anti-inflammatory herbs such as *Inula racemosa and Gymnema sylvestre* may be included in herbal remedies. These herbs have the potential to help reduce the inflammatory response that is caused by steroids and enhance insulin sensitivity in a manner that is distinct from the approach that is used for the treatment of chronic diabetic mellitus (DM) [51, 48].

### 5.9 Synthetic Medication Versus Herbal Drug on Glucocorticoid Induces Diabetes

Thiazolidinediones, often known as TZDs, have the potential to enhance diabetes-related parameters by inhibiting the pathways that lead to insulin resistance generated by glucocorticoids and by correcting the negative effects that glucocorticoids have on the activity of β-cells [67]. However, a great number of unfavourable effects are also observed, such as edema as well as congestive heart failure. Peripheral edema was seen in 33% of the diabetes patients in the pioglitazone group and 21% of the patients in the rosiglitazone group [68].

Herbal medicine, also known as phytomedicine or botanical medicine, utilizes plants for therapeutic purposes. Comparatively, the use of herbal medicine for the management and control of conditions, such as diabetes, has a longer history than the use of conventional medicine [69]. Because the potent components present in herbs have synergistic effects, using plants instead of synthetic medications may be more beneficial [70].

Herbal medications are less expensive and have fewer negative effects than chemical drugs, such as cellulose, and are becoming more popular owing to their unique properties, which include cost-effectiveness, biocompatibility, non-toxicity, and biodegradability. Cellulose nanocrystals are natural, non-toxic, and neutral substances that may have a rod-like or spherical form. They are isolated from jujube seeds; these nanocrystals activate glucokinase, significantly reducing blood glucose levels, and are effective in diabetes management [71].

### 5.10 Potential Benefits and Limitations of Herbal Therapies for Diabetes Management

Utilizing plants to manage diabetes is a widespread chronic condition that affects many people globally. This condition is characterized by high blood sugar levels caused by impaired insulin production or function. Conventional medicine has effective treatments available, but there are individuals who opt for herbal alternatives.

Much research has looked at the effects of individual herbal species on SIDM, but there is a rising interest in investigating the possible advantages of combining various herbs. This strategy may provide various benefits:

- Combining herbs may result in synergistic effects, where the combined impact outweighs the separate ones [72].
- Herbs may target various pathways in SIDM, offering a more holistic therapy strategy [73].
- Combining herbs with complementary characteristics may lessen the chance of harmful effects from individual plants.

#### 5.10.1 Advantages of Herbal Therapies

1. **Natural Approach:** Herbal remedies offer a natural approach to managing one's health, which some individuals may prefer.
2. **Potential Blood Sugar Benefits:** Some herbs, such as cinnamon and berberine, have demonstrated potential in lowering blood sugar levels in certain studies.
3. **Fewer Side Effects (Potentially):** Herbal remedies might have fewer side effects than conventional medications, although their effectiveness must be considered.
4. **Complementary Treatment:** Herbs, when used alongside conventional medicine, may provide additional benefits like improved blood pressure or cholesterol management [74-76].

#### 5.10.2 Limitations of Herbal Therapies

The available evidence on the efficacy of herbs in managing diabetes is generally limited and further investigation is necessary.

1. Herbal remedies often lack standardization, making it challenging to establish a safe and effective dosage.
2. Herbal remedies may interact with conventional medications, potentially leading to adverse effects. It is essential to communicate openly with your healthcare provider before starting any herbal treatment.
3. Some herbs can be toxic or have unintended side effects when taken in high doses or for extended periods.
4. Unlike pharmaceutical drugs, herbal supplements are not subject to the same level of regulation, which raises concerns about their purity and quality.

#### 5.10.3 Herbal Modulation of Insulin Signaling Pathways

Regulating insulin signalling pathways is important for controlling blood sugar levels. Depending on the particular plant extract or ingredient, the key goals that herbal treatments aim to achieve within the insulin signalling pathway might be different from one another. The target could potentially be a protein or enzyme involved in the pathway, like insulin receptors or glucose transporters. Research has shown that some plant extracts can improve insulin receptor sensitivity or boost glucose transporters' translocation to the cell membrane.

In other cases, the active ingredient in an herbal remedy could be a gene that produces a protein that plays a role in the insulin signalling process. Genes located near this target gene may also affect its expression and function [77]. Glucocorticoids that can influence glucose metabolism. These hormones may interact with regulatory elements in neighbouring genes to achieve the desired effect by modulating the expression of the target gene [78]. The interaction between the target gene and nearby genes may influence the overall effectiveness of herbal drugs in modifying insulin signalling pathways [77].

Further research is necessary to gain a deeper comprehension of the specific molecular pathways responsible for the influence of herbal drugs on insulin signalling. On the other hand, the information that is now available indicates that these drugs have the potential to provide innovative treatment options for illnesses that are associated with insulin resistance and type 2 diabetes [79].

#### 5.10.4 Specific phytochemical groups and their effects in Modifying Metabolic Pathways

Flavonoids, plant-based compounds known for their antioxidant and anti-inflammatory properties, have shown promising effects in treating Diabetes SIDM. Quercetin and kaempferol, found in foods like onions, apples, and berries, have been shown to improve insulin sensitivity and reduce inflammation by inhibiting pro-inflammatory cytokines. Kaempferol, present in broccoli, kale, and tea, has been shown to activate AMPK, a key regulator of cellular energy homeostasis, potentially counteracting metabolic disturbances caused by steroid use [45, 80]. Alkaloids, nitrogen-containing organic compounds found in plants like Berberine, have been shown to regulate glucose metabolism and may help mitigate some of the adverse effects of steroid use on lipid metabolism [74]. Terpenoids, a diverse class of naturally occurring organic chemicals, have shown potential in modulating insulin signaling pathways. Some terpenoids, such as those found in bitter melon (*Momordica charantia*), have been shown to activate PPAR-γ, a nuclear receptor that plays a crucial role in glucose and lipid metabolism [81].

### 5.11 Mechanisms of Action:

**Fig. (10):**
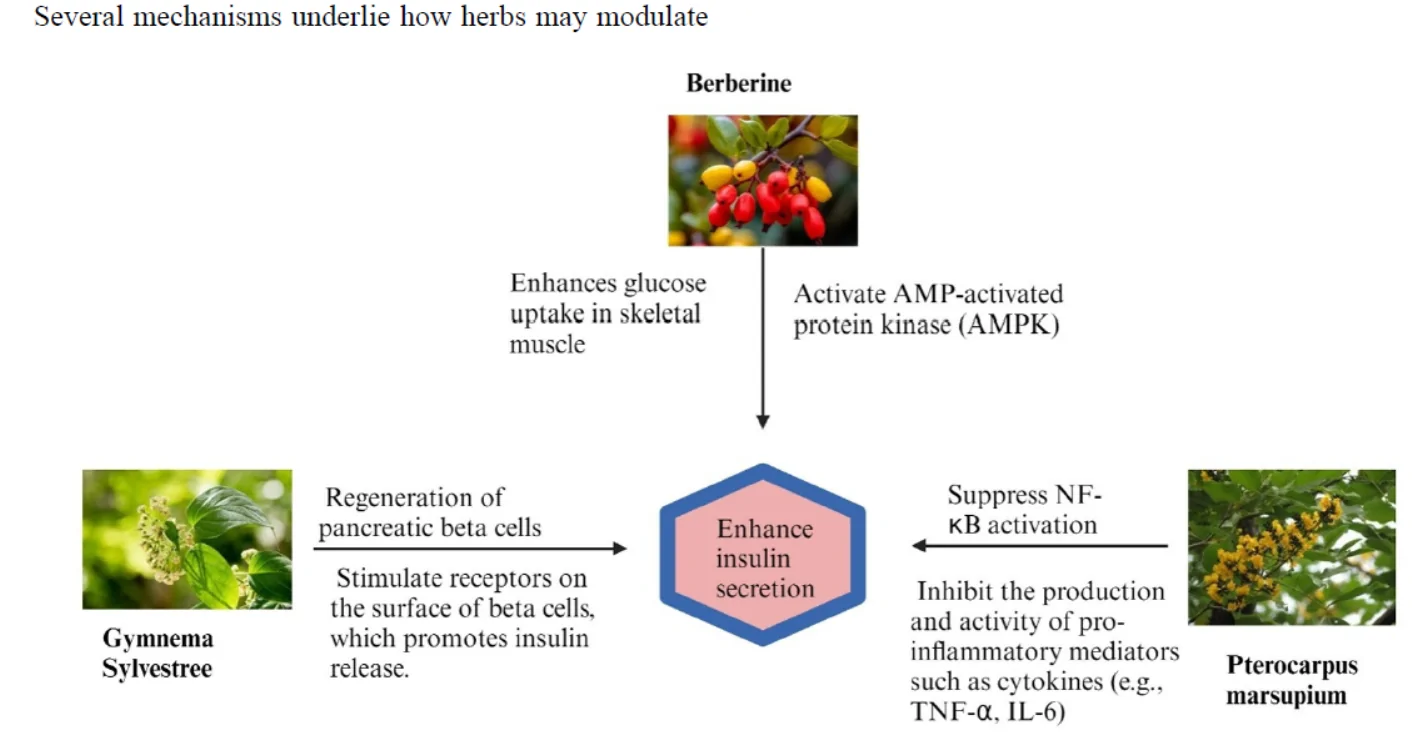
With several mechanisms, herbs can modulate insulin signaling, enhancing glucose uptake and regulating blood sugar levels effectively.

Several mechanisms underlie how herbs may modulate insulin signaling (Fig **10**).

1. **Improved Insulin Sensitivity:** Herbs containing particular polyphenols, such as berberine, can boost insulin sensitivity. They accomplish this by activating enzymes such as AMPK, which play an important role in glucose uptake into cells [74].
2. **Insulin Secretion Regulation:** Certain herbs, such as *Gymnema Sylvestre*, have been shown to regulate insulin secretion by pancreatic beta cells. This regulation helps to maintain healthy blood sugar levels by keeping a proper balance of insulin release [82].
3. **Modulation of Inflammatory Pathways:** Chronic inflammation is closely associated with insulin resistance. Herbs like *Pterocarpus marsupium* have anti-inflammatory effects that may indirectly improve insulin signaling by lowering inflammation throughout the body [83, 84]. Another plant, *Citrus medica*, has anti-inflammatory properties that help decrease inflammation and increase insulin sensitivity [85]. Research also suggests that *Citrus medica* may contain compounds that enhance the solubility and bioavailability of cholesterol-lowering properties [86]. Hence, high levels of low-density lipoprotein (LDL) cholesterol can lead to insulin resistance. Therefore, *Citrus medica* may offer a natural approach to managing diabetes [87].

Understanding how these herbs alter insulin signaling pathways provides exciting opportunities for creating complementary therapeutics for diabetes.

### 5.12 Challenges of Studying Herbal-Insulin Signaling Interactions in SIDM

The current state of the art in herbal remedy standardization has substantial hurdles because of the inherent heterogeneity of plant components. Nonetheless, progress has been made in producing standardized extracts for certain botanical species. Some herbal species are more adaptable to cultivation and extraction processes that fulfill standardization requirements [88]. Several factors contribute to this, including:

- **Limited Research:** Studies specifically investigating how herbs interact with insulin signaling pathways in SIDM are scarce. Most research focuses on general type 2 diabetes, and extrapolating those findings to SIDM requires caution.
- **Glucocorticoid Influence:** Glucosteroids have complex effects on various aspects of metabolism. Isolating the specific effects of herbs on insulin signaling within this context can be challenging.
- **Standardization Issues:** Herbal remedies can vary significantly in their active ingredient content. Studies with standardized extracts are needed for reliable results.

### 5.13 The Future of Herbal Therapies

Research into herbal modulation of insulin signaling pathways holds promise for developing complementary or alternative therapies for diabetes management. However, more rigorous studies with standardized extracts and clear mechanisms of action are needed. Additionally, safety profiles and potential interactions with medications require careful evaluation because the use of plants in glucocorticoid treatment can be complex. While some herbal extracts may have compounds that exhibit anti-inflammatory or glucocorticoid-like effects, using the whole plant or extract can introduce variability in potency and efficacy due to the presence of multiple active substances.

### 5.14 Safety Considerations and Potential Interactions

The link between diabetes and hyperglycemia is intricate and complicated. Steroid-induced diabetes, a common side effect of glucocorticoid medication, is characterized by hyperglycemia due to increased insulin resistance and decreased insulin production [67, 89]. Herbal extracts have shown promise in treating this illness *via* a variety of processes. These include modifying insulin signalling pathways, reducing steroid-induced inflammation, demonstrating antioxidant properties, boosting glucose absorption in peripheral tissues, and reducing cortisol levels. However, their effectiveness and safety profiles need more exploration, especially in terms of possible interactions with steroid drugs. Standardization and rigorous clinical studies are necessary to fully validate the potential of herbal drugs in this context. Studies have shown that herbal preparations such as *Gymnema sylvestre* [51] and *Pterocarpus marsupium* make insulin work better and control insulin release, which lessens the effects of steroids on high blood sugar [55].

Safety concerns and possible interactions must be taken into account while using herbs to treat steroid-induced diabetes. Although herbs may present a promising approach to blood sugar control, care needs to be taken because of the possibility of negative side effects and drug interactions. First and foremost, seeking medical advice is imperative before introducing herbs into a treatment plan, particularly if you are already on other drugs. Certain herbs can make underlying medical issues worse or interfere with diabetic medicines. People should also be aware of any allergic responses or negative consequences that may arise from using particular plants.

**Ginseng:** Studies on ginseng and diabetes are inconclusive. Some research suggests potential benefits like improved insulin sensitivity, while others show no significant effect. Importantly, interactions with medications like blood sugar-lowering drugs have been documented, potentially leading to hypoglycemia [90].

**Fenugreek:** Studies suggest fenugreek may improve glycemic control in type 2 diabetes, but the evidence for steroid-induced diabetes is sparse. Similar to ginseng, interactions with blood sugar-lowering medications are a concern, requiring caution and close monitoring [91].

**Bitter Melon:** Studies on bitter melon show some promise in lowering blood sugar in type 2 diabetes. However, for steroid-induced diabetes, more research is needed. Potential interactions with medications and blood sugar-lowering effects necessitate caution [92].

#### 5.14.1 Herbs that can interact with steroids:

**St. John's Wort:** This herb is known to interact with various medications, including steroids. Studies suggest it can induce the CYP3A4 enzyme, leading to increased steroid metabolism, and potentially decreased effectiveness [93].

**Grapefruit:** Certain components in grapefruit can inhibit CYP3A4, leading to increased steroid levels and potential side effects. Grapefruit juice should be avoided by individuals taking medications that are metabolized by CYP3A4, as it can increase the amount of drug in the body and lead to adverse effects. This interaction has been well-documented [94, 95].

**Goldenseal:** While not as extensively studied, goldenseal may interact with the way some medications, including steroids, are processed by the body. More research is needed to understand specific interactions [96].

### 5.15 Ethical Concerns Surrounding Herbal Treatments for SIDM Compared to Prescribed Medications

Compared to conventionally prescribed medications, the use of herbal remedies for the treatment of SIDM poses several ethical concerns [97]. Although herbal treatments may provide a more natural approach with the potential to have fewer adverse effects, there are substantial concerns regarding standardization, quality control, and evidence of efficacy. Herbal remedies frequently lack rigorous clinical testing and may have unpredictable interactions with other medications, in contrast to prescribed medications. Additionally, there are concerns regarding the appropriate dosing of botanicals, as the active compounds can exhibit significant variability [97, 98]. Furthermore, patients may experience a delay in receiving proven effective conventional. However, prescribed medications for SIDM are also subject to ethical considerations, such as high costs, adverse effects, and access issues in developing countries. Ultimately, before we can ethically recommend herbal treatments for SIDM as alternatives to conventional medicine, we need to conduct additional research to determine their safety and efficacy. Meanwhile, it is crucial to fully inform patients about the potential benefits and drawbacks of both approaches.

## CONCLUSION

Steroids are a class of medications widely used to address a variety of medical issues. Glucocorticoids, a type of steroid, can lead to diabetes, which is a frequent and sometimes dangerous side effect. Despite their anti-inflammatory and immunosuppressive properties, which often lead to their prescription, glucocorticoids can result in the development or exacerbation of diabetes. This can be a significant problem, as medications based on Western medical principles or allopathic medicine can be ineffective, carry the risk of harmful consequences, and can be prohibitively expensive, particularly in developing countries. Therefore, an alternative approach for treating steroid-induced diabetes is highly desirable. Plant-derived chemicals, specifically herbal remedies, are a promising option because they are easily accessible and do not require the same level of pharmaceutical production as synthetic treatments. The herbal remedies covered in this study have demonstrated considerable clinical and pharmacological efficacy in controlling diabetes. Additionally, herbal remedies tend to have a higher potency and fewer negative side effects than synthetic antidiabetic medications. Many herbal remedies have been used for centuries in traditional medicine and have been scientifically validated for their effectiveness in controlling diabetes. This makes them a valuable alternative for those who prefer natural treatments to synthetic ones.

## Figures and Tables

**Fig. (1):**
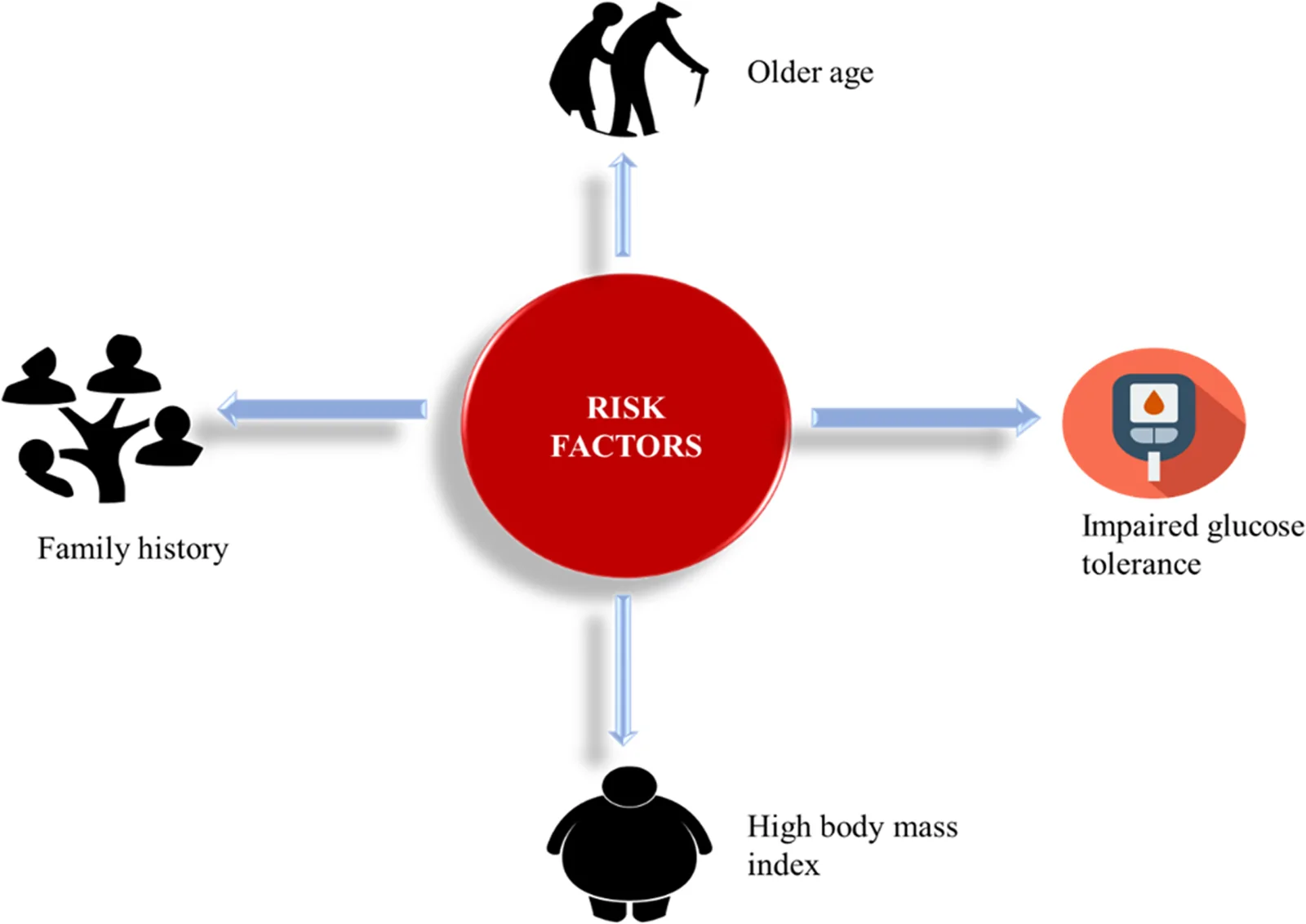
Risk factor for the progression of steroid-induced diabetes mellitus.

**Fig. (2):**
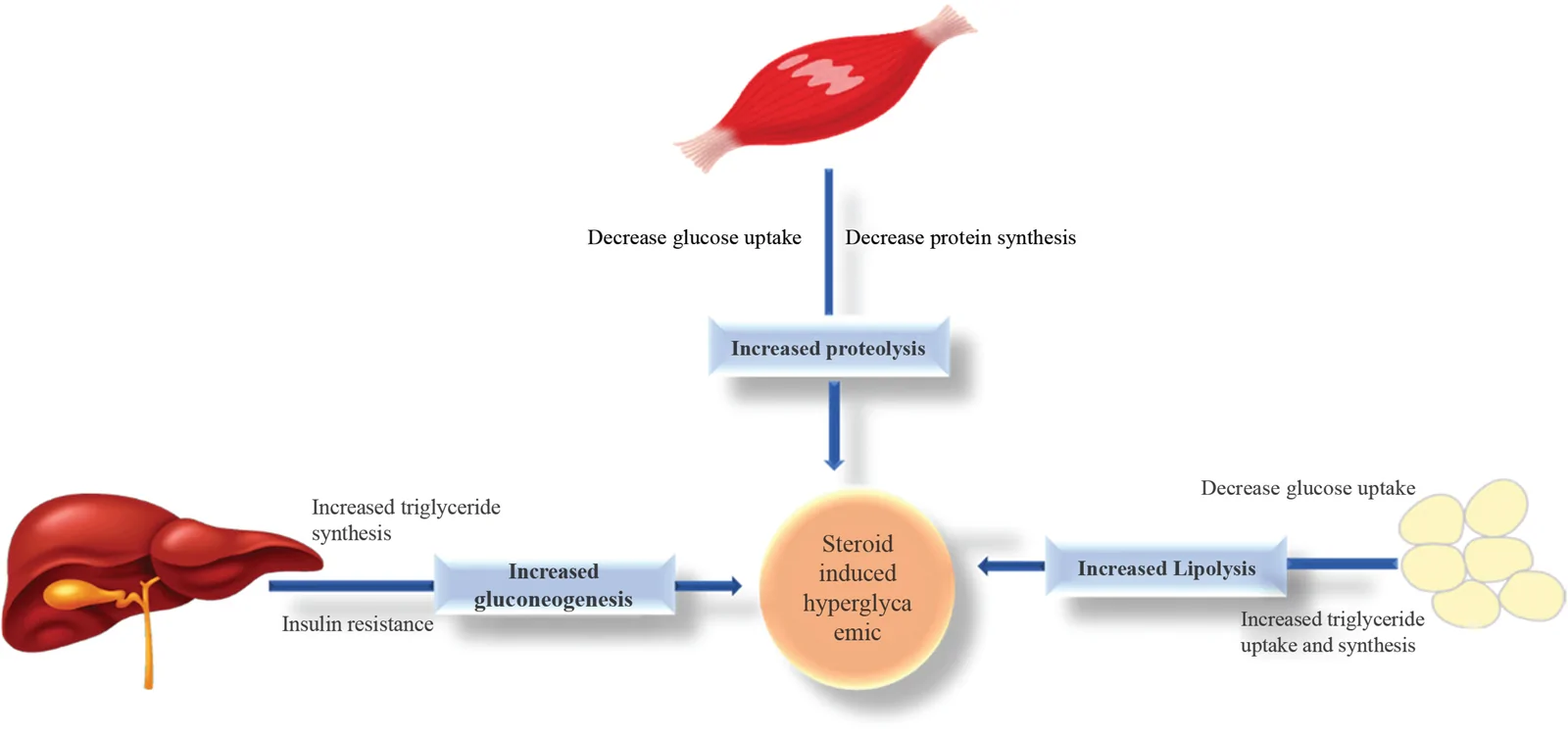
Pathophysiology of steroid-induced diabetes.

**Fig. (4):**
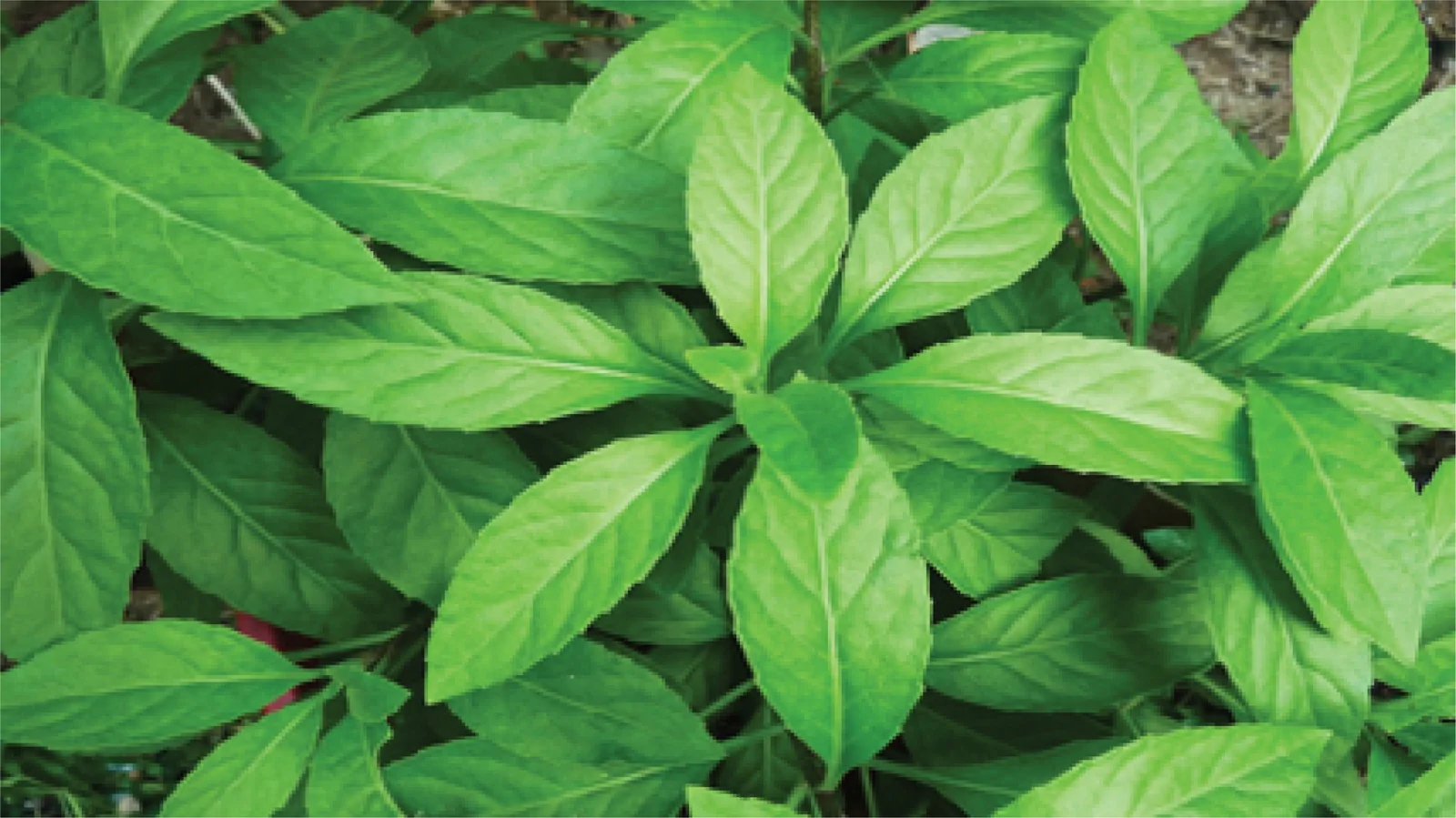
The herbal plant of *Gynura procumbens*.

**Table 1:** Steroid-induced diabetes and hyperglycaemia.

• Steroid-induced diabetes
Individuals who develop diabetes as a result of steroid medication without having previously been diagnosed with diabetes.
• Steroid-induced hyperglycaemia
A more general term for a rise in blood glucose levels caused by steroid treatment. This includes people who already have diabetes and have a worsening of their glycaemic control.
